# (*Z*)-3-Ferrocenyl-2-(4-pyridyl)­propene­nitrile

**DOI:** 10.1107/S1600536808023106

**Published:** 2008-07-26

**Authors:** Xue-Qun Fu, Wei Wang

**Affiliations:** a Ordered Matter Science Research Center, Southeast University, Nanjing 210096, People’s Republic of China

## Abstract

In the title compound, [Fe(C_5_H_5_)(C_13_H_9_N_2_)], the pyridine ring makes a dihedral angle of 9.91 (17)° with the substituted cyclo­penta­dienyl ring and the double bond adopts a *Z* configuration. In the crystal structure, inter­molecular C—H⋯N hydrogen bonds link the molecules into a one-dimensional chain in the *a*+*c* direction.

## Related literature

For related literature, see: Dupont *et al.* (2005 ▶); Shao *et al.* (2005 ▶).
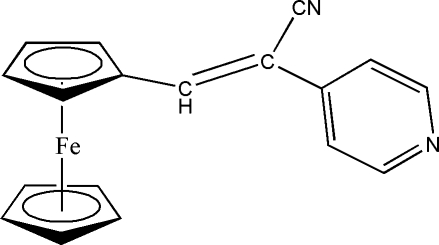


## Experimental

### 

#### Crystal data

[Fe(C_5_H_5_)(C_13_H_9_N_2_)]
*M* *_r_* = 314.16
Monoclinic, 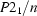

*a* = 11.520 (2) Å
*b* = 6.0650 (15) Å
*c* = 20.421 (5) Å
β = 91.194 (18)°
*V* = 1426.5 (6) Å^3^
*Z* = 4
Mo *K*α radiation
μ = 1.05 mm^−1^
*T* = 293 (2) K
0.40 × 0.35 × 0.10 mm

#### Data collection

Rigaku SCXmini diffractometer
Absorption correction: multi-scan (*CrystalClear*; Rigaku, 2005 ▶) *T*_min_ = 0.779, *T*_max_ = 1.000 (expected range = 0.701–0.900)
13960 measured reflections
3229 independent reflections
2543 reflections with *I* > 2σ(*I*)
*R*_int_ = 0.051

#### Refinement

*R*[*F* ^2^ > 2σ(*F* ^2^)] = 0.049
*wR*(*F* ^2^) = 0.122
*S* = 1.05
3229 reflections
190 parameters
H-atom parameters constrained
Δρ_max_ = 0.36 e Å^−3^
Δρ_min_ = −0.46 e Å^−3^

### 

Data collection: *CrystalClear* (Rigaku, 2005 ▶); cell refinement: *CrystalClear*; data reduction: *CrystalClear*; program(s) used to solve structure: *SHELXS97* (Sheldrick, 2008 ▶); program(s) used to refine structure: *SHELXL97* (Sheldrick, 2008 ▶); molecular graphics: *SHELXTL* (Sheldrick, 2008 ▶); software used to prepare material for publication: *SHELXTL*.

## Supplementary Material

Crystal structure: contains datablocks I, global. DOI: 10.1107/S1600536808023106/kj2092sup1.cif

Structure factors: contains datablocks I. DOI: 10.1107/S1600536808023106/kj2092Isup2.hkl

Additional supplementary materials:  crystallographic information; 3D view; checkCIF report

## Figures and Tables

**Table 1:** Hydrogen-bond geometry (Å, °)

*D*—H⋯*A*	*D*—H	H⋯*A*	*D*⋯*A*	*D*—H⋯*A*
C14—H14*A*⋯N2^i^	0.98	2.57	3.476 (4)	153

Symmetry code: (i) 
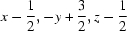
.

**Fig. 1.**
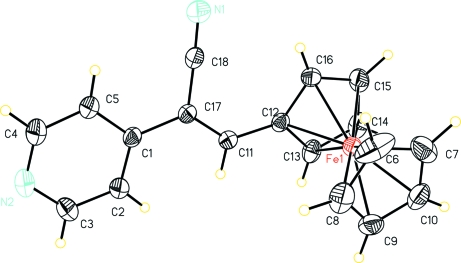
Perspective view of the title compound, showing the atom-numbering scheme. Displacement ellipsoids are drawn at the 30% probability level, and all H atoms have been omitted for clarity.

**Fig. 2.**
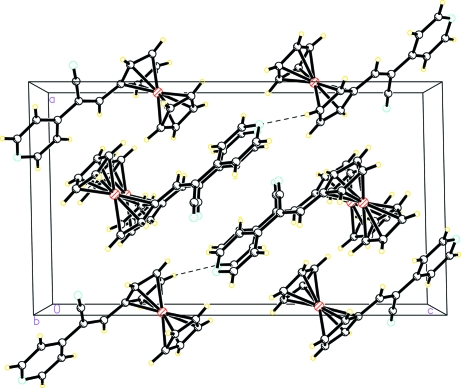
Packing of the title compound, viewed along the b axis. Dashed lines indicate C—H···N hydrogen-bond interactions.

## supplementary crystallographic information

### Comment

The chemistry of ferrocene has received much attention not only because of its exquisite structure but also its potential applications in many fields such as non-linear optical materials, catalyst and magnetoelectric materials (Dupont *et al.*, 2005). The molecular structure of the title compound is shown in Fig. 1. The C11═C17 bond exhibits a *Z* configuration and the dihedral angle between the pyridine ring and the substituted cyclopentadienyl ring is 9.91 (17)°. The two cyclopentadienyl rings are nearly parallel with the dihedral angle of 3.3 (2)°. The Fe···*Cg*1 and Fe···*Cg*2 distances are 1.6611 (19) Å and 1.6563 (15) Å respectively and the *Cg*1···Fe···*Cg*2 angle is 176.18 (8), where *Cg*1 and *Cg*2 are the centroids of the unsubstituted and substituted Cp rings. Weak intermolecular C—H···N interactions are also found in the crystal structure, which link the compound into one-dimensional chain running in the a+c direction. Similar C—H···N hydrogen bonds in a ferrocene derivative were communicated by Shao *et al.* (2005).

### Experimental

1 ml pyrrolidine was added to the mixture of formylferrocene (2.15 g, 0.01 mol) and 4-pyridineacetonitrile (1.18 g, 0.01 mol) in dichloromethane (100 ml). The mixture was stirred at room temperature for 5 h. After removing the solvent under reduced pressure, the residue was collected and dried in a vacuum desiccator. This crude product was purified by chromatography on silica gel, with petroleum ether and ethyl acetate (10:1 *v*/*v*) as eluent. Brownish red single crystals suitable for X-ray analysis were obtained by slow evaporation of ethanol at room temperature after 24 h.

### Refinement

Positional parameters of all the H atoms were calculated geometrically and were allowed to ride on the C atoms to which they are bonded, with *U*_iso_(H) = 1.2*U*_eq_(C).

### Crystal data

**Table:**

[Fe(C_5_H_5_)(C_13_H_9_N_2_)]	*F*_000_ = 648
*M**_r_* = 314.16	*D*_x_ = 1.463 Mg m^−^^3^
Monoclinic, *P*2_1_/*n*	Mo *K*α radiation λ = 0.71073 Å
Hall symbol: -P 2yn	Cell parameters from 3207 reflections
*a* = 11.520 (2) Å	θ = 2.6–27.5º
*b* = 6.0650 (15) Å	µ = 1.05 mm^−^^1^
*c* = 20.421 (5) Å	*T* = 293 (2) K
β = 91.194 (18)º	Prism, red brown
*V* = 1426.5 (6) Å^3^	0.40 × 0.35 × 0.10 mm
*Z* = 4	

### Data collection

**Table:**

Rigaku SCXmini diffractometer	3229 independent reflections
Radiation source: fine-focus sealed tube	2543 reflections with *I* > 2σ(*I*)
Monochromator: graphite	*R*_int_ = 0.051
Detector resolution: 13.6612 pixels mm^-1^	θ_max_ = 27.5º
*T* = 293(2) K	θ_min_ = 3.5º
ω scans	*h* = −14→14
Absorption correction: multi-scan(CrystalClear; Rigaku, 2005)	*k* = −7→7
*T*_min_ = 0.779, *T*_max_ = 1.000	*l* = −26→26
13960 measured reflections	

### Refinement

**Table:**

Refinement on *F*^2^	Secondary atom site location: difference Fourier map
Least-squares matrix: full	Hydrogen site location: inferred from neighbouring sites
*R*[*F*^2^ > 2σ(*F*^2^)] = 0.049	H-atom parameters constrained
*wR*(*F*^2^) = 0.123	*w* = 1/[σ^2^(*F*_o_^2^) + (0.0583*P*)^2^ + 0.4315*P*] where *P* = (*F*_o_^2^ + 2*F*_c_^2^)/3
*S* = 1.05	(Δ/σ)_max_ < 0.001
3229 reflections	Δρ_max_ = 0.36 e Å^−^^3^
190 parameters	Δρ_min_ = −0.46 e Å^−^^3^
Primary atom site location: structure-invariant direct methods	Extinction correction: none

### Special details

**Table:**

Geometry. All e.s.d.'s (except the e.s.d. in the dihedral angle between two l.s. planes) are estimated using the full covariance matrix. The cell e.s.d.'s are taken into account individually in the estimation of e.s.d.'s in distances, angles and torsion angles; correlations between e.s.d.'s in cell parameters are only used when they are defined by crystal symmetry. An approximate (isotropic) treatment of cell e.s.d.'s is used for estimating e.s.d.'s involving l.s. planes.
Refinement. Refinement of *F*^2^ against ALL reflections. The weighted *R*-factor *wR* and goodness of fit *S* are based on *F*^2^, conventional *R*-factors *R* are based on *F*, with *F* set to zero for negative *F*^2^. The threshold expression of *F*^2^ > 2σ(*F*^2^) is used only for calculating *R*-factors(gt) *etc*. and is not relevant to the choice of reflections for refinement. *R*-factors based on *F*^2^ are statistically about twice as large as those based on *F*, and *R*- factors based on ALL data will be even larger.

### Fractional atomic coordinates and isotropic or equivalent isotropic displacement parameters (Å^2^)

**Table:**

	*x*	*y*	*z*	*U*_iso_*/*U*_eq_	
Fe1	0.51801 (3)	0.90867 (6)	0.199529 (18)	0.03900 (15)	
C12	0.4765 (2)	0.8530 (5)	0.29581 (12)	0.0390 (6)	
C11	0.5556 (2)	0.8741 (5)	0.35128 (13)	0.0414 (6)	
H11A	0.6106	0.7624	0.3553	0.050*	
C17	0.5622 (2)	1.0306 (5)	0.39804 (12)	0.0383 (6)	
C18	0.4829 (3)	1.2149 (5)	0.39637 (13)	0.0468 (7)	
C16	0.3975 (2)	1.0073 (6)	0.26614 (14)	0.0473 (7)	
H16A	0.3840	1.1590	0.2807	0.057*	
C15	0.3415 (3)	0.9013 (6)	0.21203 (16)	0.0567 (8)	
H15A	0.2832	0.9684	0.1826	0.068*	
C1	0.6478 (2)	1.0312 (5)	0.45305 (12)	0.0397 (6)	
C10	0.5885 (3)	0.8562 (7)	0.10971 (17)	0.0654 (10)	
H10A	0.5645	0.7404	0.0788	0.079*	
C5	0.6533 (3)	1.2051 (5)	0.49758 (14)	0.0540 (8)	
H5A	0.6007	1.3211	0.4941	0.065*	
C2	0.7280 (3)	0.8635 (5)	0.46294 (15)	0.0526 (8)	
H2A	0.7274	0.7411	0.4355	0.063*	
C13	0.4691 (3)	0.6536 (5)	0.25760 (14)	0.0496 (7)	
H13A	0.5138	0.5185	0.2658	0.060*	
C7	0.5411 (3)	1.0641 (8)	0.1125 (2)	0.0766 (13)	
H7A	0.4800	1.1236	0.0835	0.092*	
N2	0.8151 (3)	1.0472 (6)	0.55546 (14)	0.0719 (9)	
C9	0.6729 (3)	0.8351 (7)	0.15696 (17)	0.0628 (9)	
H9A	0.7189	0.7019	0.1654	0.075*	
N1	0.4213 (3)	1.3642 (5)	0.39676 (15)	0.0733 (9)	
C8	0.6822 (3)	1.0285 (8)	0.19148 (19)	0.0735 (11)	
H8A	0.7371	1.0587	0.2277	0.088*	
C4	0.7369 (3)	1.2044 (6)	0.54673 (17)	0.0683 (10)	
H4A	0.7384	1.3226	0.5758	0.082*	
C14	0.3852 (3)	0.6842 (6)	0.20690 (15)	0.0584 (9)	
H14A	0.3621	0.5750	0.1737	0.070*	
C6	0.5998 (4)	1.1787 (6)	0.1642 (3)	0.0914 (16)	
H6A	0.5877	1.3322	0.1774	0.110*	
C3	0.8088 (3)	0.8783 (7)	0.51349 (18)	0.0696 (10)	
H3A	0.8621	0.7641	0.5187	0.084*	

### Atomic displacement parameters (Å^2^)

**Table:**

	*U*^11^	*U*^22^	*U*^33^	*U*^12^	*U*^13^	*U*^23^
Fe1	0.0337 (2)	0.0464 (3)	0.0369 (2)	−0.00351 (17)	0.00162 (15)	−0.00326 (17)
C12	0.0347 (13)	0.0474 (15)	0.0352 (13)	−0.0041 (11)	0.0052 (11)	−0.0042 (11)
C11	0.0385 (14)	0.0476 (16)	0.0381 (14)	0.0034 (12)	0.0023 (11)	−0.0023 (12)
C17	0.0381 (13)	0.0438 (15)	0.0331 (13)	0.0010 (11)	0.0043 (11)	−0.0007 (11)
C18	0.0560 (17)	0.0494 (17)	0.0350 (14)	0.0059 (14)	−0.0010 (13)	−0.0052 (12)
C16	0.0337 (13)	0.0643 (18)	0.0439 (16)	0.0046 (14)	0.0025 (12)	−0.0034 (14)
C15	0.0327 (14)	0.090 (3)	0.0468 (17)	−0.0094 (16)	−0.0024 (13)	−0.0015 (16)
C1	0.0403 (14)	0.0479 (15)	0.0311 (13)	−0.0009 (12)	0.0054 (11)	−0.0001 (11)
C10	0.067 (2)	0.085 (3)	0.0450 (18)	−0.007 (2)	0.0142 (17)	−0.0103 (17)
C5	0.0583 (18)	0.0573 (19)	0.0461 (16)	0.0090 (15)	−0.0074 (14)	−0.0147 (14)
C2	0.0554 (18)	0.0564 (18)	0.0457 (16)	0.0118 (14)	−0.0065 (14)	−0.0081 (14)
C13	0.0584 (18)	0.0493 (16)	0.0412 (15)	−0.0135 (14)	0.0041 (14)	−0.0039 (13)
C7	0.051 (2)	0.108 (4)	0.071 (2)	0.008 (2)	0.0099 (18)	0.045 (2)
N2	0.076 (2)	0.089 (2)	0.0502 (16)	0.0058 (18)	−0.0193 (15)	−0.0102 (16)
C9	0.0493 (18)	0.079 (2)	0.061 (2)	0.0138 (17)	0.0159 (16)	0.0095 (18)
N1	0.091 (2)	0.0681 (19)	0.0603 (18)	0.0309 (18)	−0.0086 (17)	−0.0103 (15)
C8	0.0479 (19)	0.114 (3)	0.058 (2)	−0.036 (2)	0.0079 (17)	−0.008 (2)
C4	0.073 (2)	0.074 (2)	0.057 (2)	0.006 (2)	−0.0164 (18)	−0.0215 (19)
C14	0.0529 (18)	0.077 (2)	0.0453 (17)	−0.0288 (17)	0.0042 (14)	−0.0083 (16)
C6	0.113 (4)	0.0426 (19)	0.121 (4)	−0.017 (2)	0.073 (3)	−0.004 (2)
C3	0.066 (2)	0.082 (2)	0.060 (2)	0.0230 (19)	−0.0200 (18)	−0.0055 (19)

### Geometric parameters (Å, °)

**Table:**

Fe1—C6	2.030 (4)	C1—C5	1.393 (4)
Fe1—C7	2.034 (3)	C10—C9	1.361 (5)
Fe1—C13	2.036 (3)	C10—C7	1.376 (5)
Fe1—C8	2.037 (3)	C10—H10A	0.9800
Fe1—C10	2.046 (3)	C5—C4	1.376 (4)
Fe1—C9	2.050 (3)	C5—H5A	0.9300
Fe1—C16	2.053 (3)	C2—C3	1.378 (5)
Fe1—C15	2.055 (3)	C2—H2A	0.9300
Fe1—C14	2.056 (3)	C13—C14	1.414 (4)
Fe1—C12	2.061 (3)	C13—H13A	0.9800
C12—C16	1.431 (4)	C7—C6	1.423 (6)
C12—C13	1.441 (4)	C7—H7A	0.9800
C12—C11	1.445 (4)	N2—C4	1.321 (5)
C11—C17	1.347 (4)	N2—C3	1.336 (5)
C11—H11A	0.9300	C9—C8	1.371 (6)
C17—C18	1.443 (4)	C9—H9A	0.9800
C17—C1	1.480 (4)	C8—C6	1.421 (6)
C18—N1	1.150 (4)	C8—H8A	0.9800
C16—C15	1.422 (4)	C4—H4A	0.9300
C16—H16A	0.9800	C14—H14A	0.9800
C15—C14	1.414 (5)	C6—H6A	0.9800
C15—H15A	0.9800	C3—H3A	0.9300
C1—C2	1.386 (4)		
			
C6—Fe1—C7	40.99 (18)	C14—C15—C16	108.8 (3)
C6—Fe1—C13	163.0 (2)	C14—C15—Fe1	69.91 (17)
C7—Fe1—C13	154.67 (17)	C16—C15—Fe1	69.67 (16)
C6—Fe1—C8	40.91 (19)	C14—C15—H15A	125.6
C7—Fe1—C8	68.01 (16)	C16—C15—H15A	125.6
C13—Fe1—C8	125.95 (17)	Fe1—C15—H15A	125.6
C6—Fe1—C10	67.09 (16)	C2—C1—C5	116.1 (3)
C7—Fe1—C10	39.40 (16)	C2—C1—C17	122.7 (3)
C13—Fe1—C10	121.59 (14)	C5—C1—C17	121.2 (3)
C8—Fe1—C10	66.19 (15)	C9—C10—C7	109.6 (4)
C6—Fe1—C9	67.04 (16)	C9—C10—Fe1	70.72 (19)
C7—Fe1—C9	66.39 (15)	C7—C10—Fe1	69.8 (2)
C13—Fe1—C9	109.61 (14)	C9—C10—H10A	125.2
C8—Fe1—C9	39.21 (16)	C7—C10—H10A	125.2
C10—Fe1—C9	38.82 (14)	Fe1—C10—H10A	125.2
C6—Fe1—C16	109.02 (14)	C4—C5—C1	119.7 (3)
C7—Fe1—C16	123.12 (15)	C4—C5—H5A	120.2
C13—Fe1—C16	68.68 (14)	C1—C5—H5A	120.2
C8—Fe1—C16	126.27 (15)	C3—C2—C1	119.9 (3)
C10—Fe1—C16	157.78 (14)	C3—C2—H2A	120.0
C9—Fe1—C16	161.98 (14)	C1—C2—H2A	120.0
C6—Fe1—C15	122.00 (18)	C14—C13—C12	108.5 (3)
C7—Fe1—C15	105.45 (14)	C14—C13—Fe1	70.54 (18)
C13—Fe1—C15	67.94 (14)	C12—C13—Fe1	70.35 (16)
C8—Fe1—C15	160.24 (18)	C14—C13—H13A	125.7
C10—Fe1—C15	121.34 (14)	C12—C13—H13A	125.7
C9—Fe1—C15	156.93 (15)	Fe1—C13—H13A	125.7
C16—Fe1—C15	40.50 (12)	C10—C7—C6	107.2 (4)
C6—Fe1—C14	155.8 (2)	C10—C7—Fe1	70.8 (2)
C7—Fe1—C14	118.82 (16)	C6—C7—Fe1	69.3 (2)
C13—Fe1—C14	40.42 (13)	C10—C7—H7A	126.4
C8—Fe1—C14	159.43 (18)	C6—C7—H7A	126.4
C10—Fe1—C14	105.82 (14)	Fe1—C7—H7A	126.4
C9—Fe1—C14	122.86 (16)	C4—N2—C3	116.0 (3)
C16—Fe1—C14	68.28 (14)	C10—C9—C8	109.4 (4)
C15—Fe1—C14	40.23 (14)	C10—C9—Fe1	70.5 (2)
C6—Fe1—C12	126.17 (16)	C8—C9—Fe1	69.9 (2)
C7—Fe1—C12	161.19 (16)	C10—C9—H9A	125.3
C13—Fe1—C12	41.18 (11)	C8—C9—H9A	125.3
C8—Fe1—C12	111.72 (13)	Fe1—C9—H9A	125.3
C10—Fe1—C12	159.07 (14)	C9—C8—C6	107.6 (4)
C9—Fe1—C12	126.18 (13)	C9—C8—Fe1	70.9 (2)
C16—Fe1—C12	40.71 (11)	C6—C8—Fe1	69.3 (2)
C15—Fe1—C12	68.16 (12)	C9—C8—H8A	126.2
C14—Fe1—C12	68.51 (11)	C6—C8—H8A	126.2
C16—C12—C13	106.9 (3)	Fe1—C8—H8A	126.2
C16—C12—C11	131.2 (3)	N2—C4—C5	124.4 (3)
C13—C12—C11	121.9 (3)	N2—C4—H4A	117.8
C16—C12—Fe1	69.33 (16)	C5—C4—H4A	117.8
C13—C12—Fe1	68.48 (15)	C13—C14—C15	107.9 (3)
C11—C12—Fe1	125.30 (18)	C13—C14—Fe1	69.05 (17)
C17—C11—C12	130.0 (3)	C15—C14—Fe1	69.85 (18)
C17—C11—H11A	115.0	C13—C14—H14A	126.1
C12—C11—H11A	115.0	C15—C14—H14A	126.1
C11—C17—C18	120.2 (3)	Fe1—C14—H14A	126.1
C11—C17—C1	124.5 (3)	C8—C6—C7	106.3 (3)
C18—C17—C1	115.3 (2)	C8—C6—Fe1	69.8 (2)
N1—C18—C17	177.9 (3)	C7—C6—Fe1	69.7 (2)
C15—C16—C12	107.9 (3)	C8—C6—H6A	126.8
C15—C16—Fe1	69.83 (17)	C7—C6—H6A	126.8
C12—C16—Fe1	69.96 (15)	Fe1—C6—H6A	126.8
C15—C16—H16A	126.1	N2—C3—C2	123.9 (3)
C12—C16—H16A	126.1	N2—C3—H3A	118.1
Fe1—C16—H16A	126.1	C2—C3—H3A	118.1
			
C6—Fe1—C12—C16	76.7 (3)	C10—Fe1—C13—C12	164.24 (18)
C7—Fe1—C12—C16	35.5 (5)	C9—Fe1—C13—C12	123.00 (19)
C13—Fe1—C12—C16	−118.8 (3)	C16—Fe1—C13—C12	−37.84 (16)
C8—Fe1—C12—C16	120.9 (2)	C15—Fe1—C13—C12	−81.58 (19)
C10—Fe1—C12—C16	−159.2 (3)	C14—Fe1—C13—C12	−119.0 (3)
C9—Fe1—C12—C16	163.0 (2)	C9—C10—C7—C6	0.3 (4)
C15—Fe1—C12—C16	−37.80 (19)	Fe1—C10—C7—C6	60.1 (2)
C14—Fe1—C12—C16	−81.2 (2)	C9—C10—C7—Fe1	−59.7 (2)
C6—Fe1—C12—C13	−164.5 (2)	C6—Fe1—C7—C10	117.8 (3)
C7—Fe1—C12—C13	154.3 (4)	C13—Fe1—C7—C10	−50.0 (4)
C8—Fe1—C12—C13	−120.3 (2)	C8—Fe1—C7—C10	78.8 (3)
C10—Fe1—C12—C13	−40.4 (4)	C9—Fe1—C7—C10	36.1 (2)
C9—Fe1—C12—C13	−78.2 (2)	C16—Fe1—C7—C10	−161.3 (2)
C16—Fe1—C12—C13	118.8 (3)	C15—Fe1—C7—C10	−120.9 (2)
C15—Fe1—C12—C13	81.0 (2)	C14—Fe1—C7—C10	−79.7 (3)
C14—Fe1—C12—C13	37.6 (2)	C12—Fe1—C7—C10	171.8 (3)
C6—Fe1—C12—C11	−49.9 (3)	C13—Fe1—C7—C6	−167.7 (3)
C7—Fe1—C12—C11	−91.0 (5)	C8—Fe1—C7—C6	−38.9 (2)
C13—Fe1—C12—C11	114.6 (3)	C10—Fe1—C7—C6	−117.8 (3)
C8—Fe1—C12—C11	−5.7 (3)	C9—Fe1—C7—C6	−81.6 (3)
C10—Fe1—C12—C11	74.3 (5)	C16—Fe1—C7—C6	80.9 (3)
C9—Fe1—C12—C11	36.4 (3)	C15—Fe1—C7—C6	121.3 (3)
C16—Fe1—C12—C11	−126.6 (3)	C14—Fe1—C7—C6	162.6 (2)
C15—Fe1—C12—C11	−164.4 (3)	C12—Fe1—C7—C6	54.0 (5)
C14—Fe1—C12—C11	152.2 (3)	C7—C10—C9—C8	0.0 (4)
C16—C12—C11—C17	14.0 (5)	Fe1—C10—C9—C8	−59.2 (2)
C13—C12—C11—C17	−168.5 (3)	C7—C10—C9—Fe1	59.2 (2)
Fe1—C12—C11—C17	106.6 (3)	C6—Fe1—C9—C10	−81.5 (3)
C12—C11—C17—C18	−1.2 (5)	C7—Fe1—C9—C10	−36.7 (3)
C12—C11—C17—C1	179.4 (3)	C13—Fe1—C9—C10	116.4 (2)
C13—C12—C16—C15	1.4 (3)	C8—Fe1—C9—C10	−120.3 (4)
C11—C12—C16—C15	179.2 (3)	C16—Fe1—C9—C10	−162.4 (4)
Fe1—C12—C16—C15	59.8 (2)	C15—Fe1—C9—C10	36.8 (5)
C13—C12—C16—Fe1	−58.40 (19)	C14—Fe1—C9—C10	73.4 (3)
C11—C12—C16—Fe1	119.4 (3)	C12—Fe1—C9—C10	159.5 (2)
C6—Fe1—C16—C15	117.3 (3)	C6—Fe1—C9—C8	38.8 (3)
C7—Fe1—C16—C15	74.1 (3)	C7—Fe1—C9—C8	83.6 (3)
C13—Fe1—C16—C15	−80.6 (2)	C13—Fe1—C9—C8	−123.3 (2)
C8—Fe1—C16—C15	159.7 (2)	C10—Fe1—C9—C8	120.3 (4)
C10—Fe1—C16—C15	41.5 (5)	C16—Fe1—C9—C8	−42.1 (6)
C9—Fe1—C16—C15	−168.5 (4)	C15—Fe1—C9—C8	157.1 (4)
C14—Fe1—C16—C15	−37.0 (2)	C14—Fe1—C9—C8	−166.2 (2)
C12—Fe1—C16—C15	−118.8 (3)	C12—Fe1—C9—C8	−80.1 (3)
C6—Fe1—C16—C12	−123.8 (2)	C10—C9—C8—C6	−0.3 (4)
C7—Fe1—C16—C12	−167.1 (2)	Fe1—C9—C8—C6	−59.9 (2)
C13—Fe1—C16—C12	38.27 (17)	C10—C9—C8—Fe1	59.6 (3)
C8—Fe1—C16—C12	−81.4 (3)	C6—Fe1—C8—C9	−118.2 (3)
C10—Fe1—C16—C12	160.4 (4)	C7—Fe1—C8—C9	−79.1 (3)
C9—Fe1—C16—C12	−49.7 (5)	C13—Fe1—C8—C9	76.5 (3)
C15—Fe1—C16—C12	118.8 (3)	C10—Fe1—C8—C9	−36.3 (2)
C14—Fe1—C16—C12	81.86 (19)	C16—Fe1—C8—C9	165.1 (2)
C12—C16—C15—C14	−0.7 (3)	C15—Fe1—C8—C9	−153.2 (4)
Fe1—C16—C15—C14	59.1 (2)	C14—Fe1—C8—C9	34.6 (5)
C12—C16—C15—Fe1	−59.86 (19)	C12—Fe1—C8—C9	121.1 (2)
C6—Fe1—C15—C14	157.9 (2)	C7—Fe1—C8—C6	39.0 (3)
C7—Fe1—C15—C14	116.6 (2)	C13—Fe1—C8—C6	−165.3 (2)
C13—Fe1—C15—C14	−37.55 (18)	C10—Fe1—C8—C6	81.9 (3)
C8—Fe1—C15—C14	−175.7 (4)	C9—Fe1—C8—C6	118.2 (3)
C10—Fe1—C15—C14	77.0 (2)	C16—Fe1—C8—C6	−76.7 (3)
C9—Fe1—C15—C14	50.9 (4)	C15—Fe1—C8—C6	−35.1 (5)
C16—Fe1—C15—C14	−120.1 (3)	C14—Fe1—C8—C6	152.8 (4)
C12—Fe1—C15—C14	−82.1 (2)	C12—Fe1—C8—C6	−120.7 (3)
C6—Fe1—C15—C16	−82.0 (3)	C3—N2—C4—C5	−1.1 (6)
C7—Fe1—C15—C16	−123.3 (2)	C1—C5—C4—N2	0.0 (6)
C13—Fe1—C15—C16	82.6 (2)	C12—C13—C14—C15	1.1 (3)
C8—Fe1—C15—C16	−55.6 (5)	Fe1—C13—C14—C15	−59.3 (2)
C10—Fe1—C15—C16	−162.9 (2)	C12—C13—C14—Fe1	60.35 (19)
C9—Fe1—C15—C16	171.0 (3)	C16—C15—C14—C13	−0.2 (3)
C14—Fe1—C15—C16	120.1 (3)	Fe1—C15—C14—C13	58.8 (2)
C12—Fe1—C15—C16	37.99 (19)	C16—C15—C14—Fe1	−59.0 (2)
C11—C17—C1—C2	−2.4 (4)	C6—Fe1—C14—C13	−170.4 (3)
C18—C17—C1—C2	178.2 (3)	C7—Fe1—C14—C13	160.9 (2)
C11—C17—C1—C5	176.5 (3)	C8—Fe1—C14—C13	56.5 (4)
C18—C17—C1—C5	−3.0 (4)	C10—Fe1—C14—C13	120.5 (2)
C6—Fe1—C10—C9	81.4 (3)	C9—Fe1—C14—C13	81.8 (2)
C7—Fe1—C10—C9	120.4 (4)	C16—Fe1—C14—C13	−82.2 (2)
C13—Fe1—C10—C9	−82.2 (3)	C15—Fe1—C14—C13	−119.4 (3)
C8—Fe1—C10—C9	36.6 (3)	C12—Fe1—C14—C13	−38.25 (18)
C16—Fe1—C10—C9	165.7 (3)	C6—Fe1—C14—C15	−51.0 (4)
C15—Fe1—C10—C9	−164.0 (2)	C7—Fe1—C14—C15	−79.7 (2)
C14—Fe1—C10—C9	−123.2 (2)	C13—Fe1—C14—C15	119.4 (3)
C12—Fe1—C10—C9	−52.2 (5)	C8—Fe1—C14—C15	175.9 (4)
C6—Fe1—C10—C7	−39.1 (3)	C10—Fe1—C14—C15	−120.1 (2)
C13—Fe1—C10—C7	157.4 (2)	C9—Fe1—C14—C15	−158.78 (19)
C8—Fe1—C10—C7	−83.8 (3)	C16—Fe1—C14—C15	37.22 (19)
C9—Fe1—C10—C7	−120.4 (4)	C12—Fe1—C14—C15	81.2 (2)
C16—Fe1—C10—C7	45.3 (5)	C9—C8—C6—C7	0.5 (4)
C15—Fe1—C10—C7	75.5 (3)	Fe1—C8—C6—C7	−60.4 (2)
C14—Fe1—C10—C7	116.4 (3)	C9—C8—C6—Fe1	60.9 (2)
C12—Fe1—C10—C7	−172.6 (3)	C10—C7—C6—C8	−0.5 (4)
C2—C1—C5—C4	1.4 (5)	Fe1—C7—C6—C8	60.5 (3)
C17—C1—C5—C4	−177.6 (3)	C10—C7—C6—Fe1	−61.0 (2)
C5—C1—C2—C3	−1.7 (5)	C7—Fe1—C6—C8	−117.1 (3)
C17—C1—C2—C3	177.2 (3)	C13—Fe1—C6—C8	44.7 (6)
C16—C12—C13—C14	−1.5 (3)	C10—Fe1—C6—C8	−79.6 (3)
C11—C12—C13—C14	−179.6 (2)	C9—Fe1—C6—C8	−37.2 (2)
Fe1—C12—C13—C14	−60.5 (2)	C16—Fe1—C6—C8	123.9 (2)
C16—C12—C13—Fe1	58.94 (19)	C15—Fe1—C6—C8	166.8 (2)
C11—C12—C13—Fe1	−119.1 (2)	C14—Fe1—C6—C8	−156.9 (3)
C6—Fe1—C13—C14	166.5 (5)	C12—Fe1—C6—C8	81.7 (3)
C7—Fe1—C13—C14	−42.0 (4)	C13—Fe1—C6—C7	161.9 (4)
C8—Fe1—C13—C14	−158.8 (2)	C8—Fe1—C6—C7	117.1 (3)
C10—Fe1—C13—C14	−76.8 (2)	C10—Fe1—C6—C7	37.6 (2)
C9—Fe1—C13—C14	−118.0 (2)	C9—Fe1—C6—C7	79.9 (2)
C16—Fe1—C13—C14	81.1 (2)	C16—Fe1—C6—C7	−119.0 (2)
C15—Fe1—C13—C14	37.38 (19)	C15—Fe1—C6—C7	−76.1 (3)
C12—Fe1—C13—C14	119.0 (3)	C14—Fe1—C6—C7	−39.8 (5)
C6—Fe1—C13—C12	47.5 (6)	C12—Fe1—C6—C7	−161.1 (2)
C7—Fe1—C13—C12	−161.0 (3)	C4—N2—C3—C2	0.8 (6)
C8—Fe1—C13—C12	82.3 (2)	C1—C2—C3—N2	0.7 (6)

### Hydrogen-bond geometry (Å, °)

**Table:**

*D*—H···*A*	*D*—H	H···*A*	*D*···*A*	*D*—H···*A*
C14—H14A···N2^i^	0.98	2.57	3.476 (4)	153

Symmetry codes: (i) *x*−1/2, −*y*+3/2, *z*−1/2.
